# COVID-19 induced social isolation; implications for understanding social cognition in mental health

**DOI:** 10.1017/S0033291720004006

**Published:** 2020-10-08

**Authors:** A. R. Bland, J. P .Roiser, M. A. Mehta, B. J. Sahakian, T. W. Robbins, R. Elliott

**Affiliations:** 1 Department of Psychology, Manchester Metropolitan University, Manchester, UK; 2 Institute of Cognitive Neuroscience, University College London, London, UK; 3 Institute of Psychiatry, Psychology and Neuroscience, King's College London, London, UK; 4 Department of Psychiatry, University of Cambridge, Cambridge, UK; 5 Behavioural and Clinical Neuroscience Institute, University of Cambridge, Cambridge, UK; 6 Department of Psychology, University of Cambridge, Cambridge, UK; 7 Division of Neuroscience and Experimental Psychology, University of Manchester, Manchester, UK

**Keywords:** Affective bias, COVID-19, social cognition, social connectivity, social isolation

Social distancing measures to combat the spread of the severe acute respiratory syndrome-coronavirus 2 (SARS-CoV2) infections are likely to have unintended consequences on mental health and emotional wellbeing. Social isolation, loneliness and uncertainty are key risk factors for developing mental health problems and pose a significant concern for the long-term consequences of social distancing (Vatansever, Wang, & Sahakian, 2020). Nevertheless, this pandemic has illuminated the struggle of many people with mental health disorders, who live socially disconnected and isolated lives every day, long before the emergence of coronavirus 2019 (COVID-19) and societal ‘lockdown’.

Social integration has been found to be robustly linked to social cognitive ability; the mental operations needed to perceive, interpret and process information for adaptive social interactions (Green, Horan, & Lee, 2019). Without the ability to interpret emotional facial expressions in others and understand subtle social cues, social integration and maintaining social support networks is problematic. A fundamental question remains as to whether social cognition deficits are inherent vulnerability markers of mental health problems, whereby people with impaired social cognitive skills have difficulty with forming normal social support networks resulting in withdrawal and loneliness, or whether they are a secondary consequence of prolonged periods of isolation and poor social connections resulting from mental health symptoms.

Social distancing measures have presented a unique opportunity to examine the effects of social isolation on people without prior mental health disorders in order to ascertain whether social isolation has a detrimental impact on social cognitive ability. This will inform the extent to which social cognitive deficits are attributable to reduced social contact. This has important implications for how we interpret social cognitive deficits in mental health disorders and inform the development of appropriate interventions. Indeed, if social isolation causes direct impairments to a particular aspect of social cognition, this suggests that preventing or reducing perceived isolation, enhancing social support and reducing loneliness may prevent the development of social cognitive deficits associated with mental health problems. This has critical implications for outcomes such as maintaining interpersonal relationships and face-to-face employment. Alternatively, if social isolation does not produce impairments in aspects of social cognition in the absence of mental health problems, it may be the case that social cognitive disruption is inherent to the pathology of mental health disorders. In this case, treatments should target symptom-specific impairments in order to improve social cognition and linked functional outcomes. This not only has important implications for understanding the effects of social isolation due to COVID-19 pandemic, but also has wider implications for understanding the interactions between social isolation, social cognition and mental health.

We examined social cognitive ability during the most stringent period of UK government enforced ‘lockdown’ (21 April to 10 May) in 107 adult participants who had not previously experienced mental health problems. Following ethical approval and with informed consent, we distributed online neuropsychological tasks assessing emotion recognition, emotional attention and cooperative behaviour to examine social cognitive ability in comparison to normative performance data obtained pre-COVID-19. For emotional facial recognition specifically, which is thought to be a robust marker of mental health disruption, we observed significantly reduced positive biases [*F*_(1306)_ = 13.46, *p* < 0.001, *η_p_^2^* = 0.09]. This was driven by significantly reduced accuracy in recognising happy faces [*F*_(1306)_ = 6.01, *p* = 0.003, *η_p_^2^* = 0.04] and significantly increased accuracy in recognising sad faces [*F*_(1306)_ = 9.92, *p* < 0.001, *η_p_^2^* = 0.07]. Interestingly, we found that affective biases during COVID-19 were related to how connected people were during lockdown with people who experienced greater disruption to their usual social network, showing the greatest decline in positive bias in emotional recognition. Our results point towards the significant impact of social distancing measures on social cognitive ability. However, it remains unclear whether the impact of social isolation is transient and returns to typical levels upon normal social connectivity resuming or whether the impact continues longer-term. Understanding these cognitive mechanisms by which enforced social isolation impacts mental health is vital. This will inform development of treatment and preventative interventions that target specific aspects of social cognition to improve functional outcomes and help inform specific targets for effective change and intervention for people at risk of debilitating mental health disorders.

## References

[ref1] Green, M. F., Horan, W. P., & Lee, J. (2019). Nonsocial and social cognition in schizophrenia: Current evidence and future directions. World Psychiatry: Official Journal of the World Psychiatric Association *(*WPA*)*, 18(2), 146–161. 10.1002/wps.20624 31059632 PMC6502429

[ref2] Vatansever, D., Wang, S., & Sahakian, B. J. (2020). Covid-19 and promising solutions to combat symptoms of stress, anxiety and depression. Neuropsychopharmacology, 0, 1–2. 10.1038/s41386-020-00791-9. PMC7425273 32792683

